# Supplementary material for: Proportion and distribution of neurotransmitter-defined cell types in the ventral tegmental area and substantia nigra pars compacta
Source: bioRxiv. 2024 Aug 29:2024.02.28.582356. Originally published 2024 Feb 28. Preprint. [Version 2] doi: 10.1101/2024.02.28.582356 (PMC10925288; doi:10.1101/2024.02.28.582356)
Supplement: Supplementary file 1 [file NIHPP2024.02.28.582356v2-supplement-1.pdf]

bioRxiv preprint doi: https://doi.org/10.1101/2024.02.28.582356; this version posted August 29, 2024. The copyright holder for this preprint (which was not certified by peer review) is the author/funder, who has granted bioRxiv a license to display the preprint in perpetuity. It is made available under aCC-BY 4.0 International license.

Supplemental Figure 1 (related to Figure 1). Schematized display of vesicular transporter-expressing neurons.

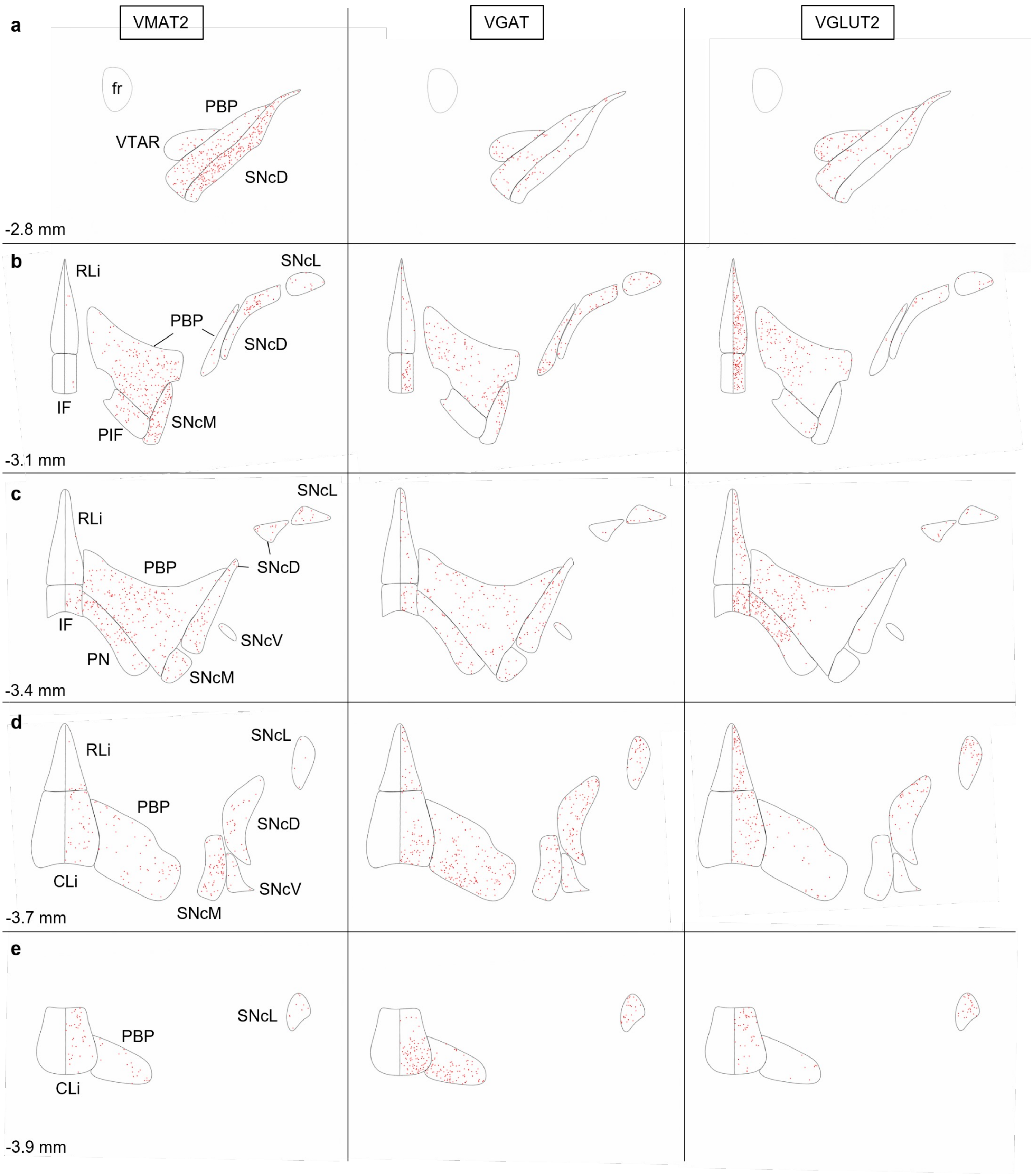

bioRxiv preprint doi: https://doi.org/10.1101/2024.02.28.582356; this version posted August 29, 2024. The copyright holder for this preprint (which was not certified by peer review) is the author/funder, who has granted bioRxiv a license to display the preprint in perpetuity. It is made available under aCC-BY 4.0 International license.

Supplemental Figure 2 (related to Figure 4). Global proportions of vesicular transporter-defined neurons in VTA and SNc displayed by sex.

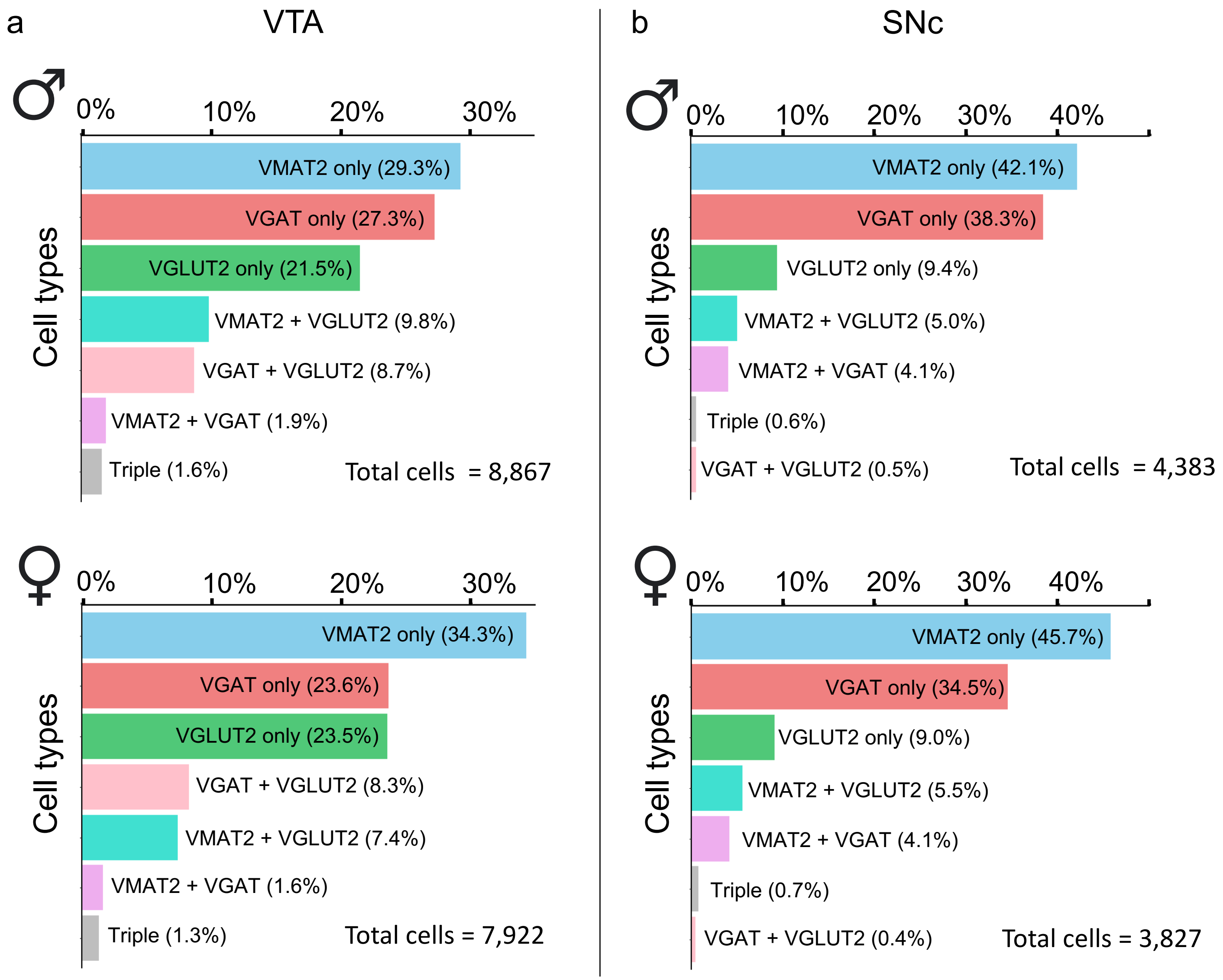

bioRxiv preprint doi: https://doi.org/10.1101/2024.02.28.582356; this version posted August 29, 2024. The copyright holder for this preprint (which was not certified by peer review) is the author/funder, who has granted bioRxiv a license to display the preprint in perpetuity. It is made available under aCC-BY 4.0 International license.

Supplemental Figure 3 (related to Figure 4). ZsG/TH co-positive neurons in VTA and SNc of VGAT-Cre reporter mice

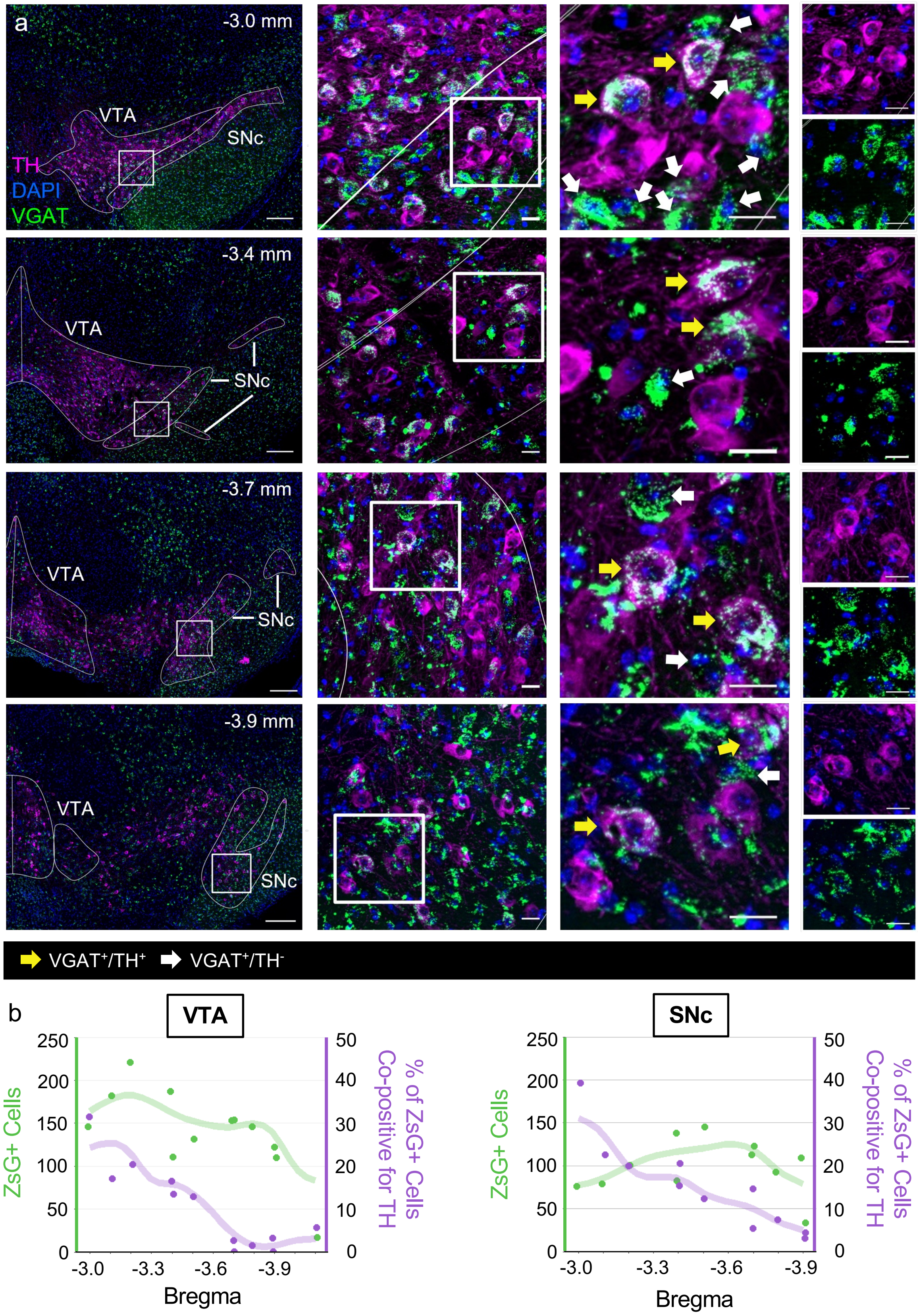

bioRxiv preprint doi: https://doi.org/10.1101/2024.02.28.582356; this version posted August 29, 2024. The copyright holder for this preprint (which was not certified by peer review) is the author/funder, who has granted bioRxiv a license to display the preprint in perpetuity. It is made available under aCC-BY 4.0 International license.

Supplemental Figure 4 (related to Figure 6). Spatial distribution of vesicular transporter-defined neurons along anterior-posterior (AP) and medial-lateral (ML) axes

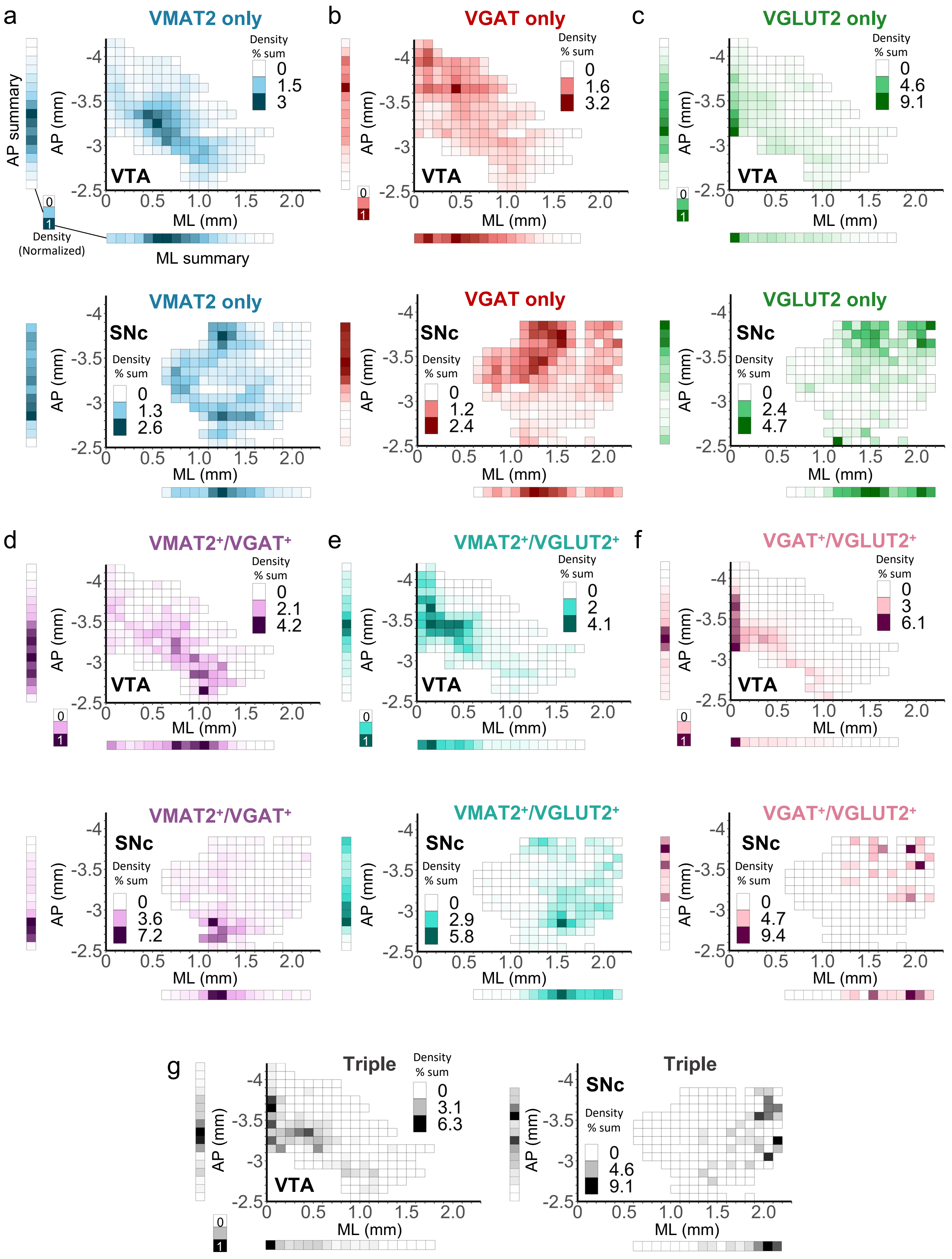

bioRxiv preprint doi: https://doi.org/10.1101/2024.02.28.582356; this version posted August 29, 2024. The copyright holder for this preprint (which was not certified by peer review) is the author/funder, who has granted bioRxiv a license to display the preprint in perpetuity. It is made available under aCC-BY 4.0 International license.

Supplemental Table 1 (related to Figure 2). Fraction of TPH2/VMAT2 copositive neurons in caudal VTA (unilateral)

Bregma (mm)	TPH2+ neuron counts	VMAT2+ neuron counts	Percentage of VMAT2+ neurons positive for TPH2	Mean percent for bregma point
**-3.6**	2	135	1.5	3.1
	2	127	1.6	
	7	94	7.4	
**-3.7**	13	109	11.9	9.0
	4	80	5	
**-3.9**	4	73	8.2	8.2
**-4.0**	2	24	8.3	7.0
	2	33	6.1	
**-4.1**	4	16	25	29.6
	4	11	36.4	
**-4.2**	13	27	48.1	48.1

bioRxiv preprint doi: https://doi.org/10.1101/2024.02.28.582356; this version posted August 29, 2024. The copyright holder for this preprint (which was not certified by peer review) is the author/funder, who has granted bioRxiv a license to display the preprint in perpetuity. It is made available under aCC-BY 4.0 International license.

Supplemental Table 2 (related to Figure 4, Supplemental Figure 3). Fraction of ZsG/TH copositive neurons in VTA and SNc of VGAT-Cre reporter mice.

Bregma (mm)	Region	ZsG+ neuron counts	ZsG+ neurons with TH	Percent of co-positive neurons
-3.0	VTA	146	46	31.5
-3.1	VTA	182	31	17
-3.2	VTA	221	45	20.4
-3.4	VTA	187	31	16.6
-3.4	VTA	111	15	13.5
-3.5	VTA	131	17	13
-3.7	VTA	154	4	2.6
-3.7	VTA	153	0	0
-3.8	VTA	146	2	1.4
-3.9	VTA	122	4	3.3
-3.9	VTA	110	0	0
-4.1	VTA	17	1	5.9
-3.0	SNc	76	30	39.5
-3.1	SNc	79	18	22.8
-3.2	SNc	101	20	19.8
-3.4	SNc	82	17	20.7
-3.4	SNc	138	21	15.2
-3.5	SNc	146	18	12.3
-3.7	SNc	123	18	14.6
-3.7	SNc	113	6	5.3
-3.8	SNc	92	7	7.6
-3.9	SNc	110	5	4.5
-3.9	SNc	34	1	2.9

bioRxiv preprint doi: https://doi.org/10.1101/2024.02.28.582356; this version posted August 29, 2024. The copyright holder for this preprint (which was not certified by peer review) is the author/funder, who has granted bioRxiv a license to display the preprint in perpetuity. It is made available under aCC-BY 4.0 International license.

Supplemental Table 3: (related to Figures 4 & 5). Cell-type counts and fractions for subregions disaggregated by sex

		n (mice)	n (sections)	Sex	VMAT2 only	VGAT only	VGLUT2 only	VMAT2$^+$/ VGAT$^+$	VMAT2$^+$/ VGLUT2$^+$	VGAT$^+$/ VGLUT2$^+$	Triple	Total
VTA global	Cumulative cell count	3	25	M	2597	2417	1906	166	871	771	139	8867
		3	23	F	2716	1871	1865	129	585	654	102	7922
		6	48	B	5313	4288	3771	295	1456	1425	241	16789
	Fraction (%)	3	25	M	29.3	27.3	21.5	1.9	9.8	8.7	1.6	-
		3	23	F	34.3	23.6	23.5	1.6	7.4	8.3	1.3	-
		6	48	B	31.6	25.5	22.5	1.8	8.7	8.5	1.4	-
PIF	Cumulative cell count	2	7	M	221	63	60	2	41	24	5	416
		2	7	F	199	46	121	4	24	20	2	416
		4	14	B	420	109	181	6	65	44	7	832
	Fraction (%)	2	7	M	53.1	15.1	14.4	0.5	9.9	5.8	1.2	-
		2	7	F	47.8	11.1	29.1	1.0	5.8	4.8	0.5	-
		4	14	B	50.5	13.1	21.8	0.7	7.8	5.3	0.8	-
PN	Cumulative cell count	2	9	M	254	211	176	10	173	26	19	869
		2	9	F	324	150	171	8	124	36	5	818
		4	18	B	578	361	347	18	297	62	24	1687
	Fraction (%)	2	9	M	29.2	24.3	20.3	1.2	19.9	3.0	2.2	-
		2	9	F	39.6	18.3	20.9	1.0	15.2	4.4	0.6	-
		4	18	B	34.3	21.4	20.6	1.1	17.6	3.7	1.4	-
PBP	Cumulative cell count	3	22	M	1824	1552	678	135	387	321	63	4960
		3	21	F	1965	1332	679	102	267	282	54	4681
		6	43	B	3789	2884	1357	237	654	603	117	9641
	Fraction (%)	3	22	M	36.8	31.3	13.7	2.7	7.8	6.5	1.3	-
		3	21	F	42.0	28.5	14.5	2.2	5.7	6.0	1.2	-
		6	43	B	39.3	29.9	14.1	2.5	6.8	6.3	1.2	-
RLi	Cumulative cell count	3	13	M	49	47	548	4	55	220	17	940
		2	11	F	8	36	473	2	26	138	10	693
		5	24	B	57	83	1021	6	81	358	27	1633
	Fraction (%)	3	13	M	5.2	5.0	58.3	0.4	5.9	23.4	1.8	-
		2	11	F	1.2	5.2	68.3	0.3	3.8	19.9	1.4	-
		5	24	B	3.5	5.1	62.5	0.4	5.0	21.9	1.7	-
IF	Cumulative cell count	2	10	M	58	39	197	5	81	98	22	500
		2	10	F	88	70	199	8	66	127	23	581
		4	20	B	146	109	396	13	147	225	45	1081
	Fraction (%)	2	10	M	11.6	7.8	39.4	1.0	16.2	19.6	4.4	-
		2	10	F	15.1	12.0	34.3	1.4	11.4	21.9	4.0	-
		4	20	B	13.5	10.1	36.6	1.2	13.6	20.8	4.2	-
VTAR	Cumulative cell count	3	6	M	95	77	104	4	12	45	0	337
		2	7	F	86	45	146	1	14	36	1	329
		5	13	B	181	122	250	5	26	81	1	666
	Fraction (%)	3	6	M	28.2	22.8	30.9	1.2	3.6	13.4	0	-
		2	7	F	26.1	13.7	44.4	0.3	4.3	10.9	0.3	-
		5	13	B	27.2	18.3	37.5	0.8	3.9	12.2	0.2	-
CLi	Cumulative cell count	3	8	M	96	428	143	6	122	37	13	845
		2	5	F	46	192	76	4	64	15	7	404
		5	13	B	142	620	219	10	186	52	20	1249
	Fraction (%)	3	8	M	11.4	50.7	16.9	0.7	14.4	4.4	1.5	-
		2	5	F	11.4	47.5	18.8	1.0	15.8	3.7	1.7	-
		5	13	B	11.4	49.6	17.5	0.8	14.9	4.2	1.6	-
SNc global	Cumulative cell count	3	22	M	1845	1681	412	179	219	22	25	4383
		3	21	F	1749	1319	345	158	212	16	28	3827
		6	43	B	3594	3000	757	337	431	38	53	8210
	Fraction (%)	3	22	M	42.1	38.4	9.4	4.1	5.0	0.5	0.6	-
		3	21	F	45.7	34.5	9.0	4.1	5.5	0.4	0.7	-
		6	43	B	43.8	36.5	9.2	4.1	5.2	0.5	0.6	-
SNcD	Cumulative cell count	3	21	M	1133	938	221	150	130	11	4	2587
		2	20	F	1126	705	230	128	156	10	11	2366
		5	41	B	2259	1643	451	278	286	21	15	4953
	Fraction (%)	3	21	M	43.8	36.3	8.5	5.8	5.0	0.4	0.2	-
		2	20	F	47.6	29.8	9.7	5.4	6.6	0.4	0.5	-
		5	41	B	45.6	33.2	9.1	5.6	5.8	0.4	0.3	-
SNcL	Cumulative cell count	3	18	M	82	330	171	9	64	11	21	688
		3	16	F	59	246	89	8	47	6	17	472
		6	34	B	141	576	260	17	111	17	38	1160
	Fraction (%)	3	18	M	11.9	48.0	24.9	1.3	9.3	1.6	3.1	-
		3	16	F	12.5	52.1	18.9	1.7	10.0	1.3	3.6	-
		6	34	B	12.2	49.7	22.4	1.5	9.6	1.5	3.3	-
SNcM	Cumulative cell count	3	15	M	571	317	18	19	12	0	0	937
		2	13	F	510	245	26	19	6	0	0	806
		5	28	B	1081	562	44	38	18	0	0	1743
	Fraction (%)	3	15	M	60.9	33.8	1.9	2.0	1.3	0	0	-
		2	13	F	63.3	30.4	3.2	2.4	0.7	0	0	-
		5	28	B	62.0	32.2	2.5	2.2	1.0	0	0	-
SNcV	Cumulative cell count	3	9	M	59	96	2	1	13	0	0	171
		2	9	F	54	123	0	3	3	0	0	183
		5	18	B	113	219	2	4	16	0	0	354
	Fraction (%)	3	9	M	34.5	56.1	1.2	0.6	7.6	0	0	-
		2	9	F	29.5	67.2	0.0	1.6	1.6	0	0	-
		5	18	B	31.9	61.9	0.6	1.1	4.5	0	0	-

bioRxiv preprint doi: https://doi.org/10.1101/2024.02.28.582356; this version posted August 29, 2024. The copyright holder for this preprint (which was not certified by peer review) is the author/funder, who has granted bioRxiv a license to display the preprint in perpetuity. It is made available under aCC-BY 4.0 International license.

Supplemental Table 4 (related to methods). Image acquisition settings.

Experiment	Label	Laser λ (nm)	Laser source	Laser Power	Gain (ms)
Slc17a6/Slc32a1/Slc18a2	DAPI	405	Diode	0.50%	611
	Slc17a6	488	Argon	0.50%	558
	Slc32a1	561	Diode-pumped solid-state	0.50%	750
	Slc18a2	633	Helium-Neon	0.50%	700
**Dual ISH and IHC**	DAPI	405	Diode	0.50%	611
	GFP	488	Argon	0.15%	558
	Slc17a6	561	Diode-pumped solid-state	0.50%	750
**TPH2**	DAPI	405	Diode	0.50%	611
	Slc18a2	488	Argon	0.40%	558
	Tph2	561	Diode-pumped solid-state	0.18%	750
	Th	633	Helium-Neon	0.50%	700
